# Acne in Lomé, Togo: clinical aspects and quality of life of patients

**DOI:** 10.1186/s12895-018-0075-z

**Published:** 2018-08-22

**Authors:** Bayaki Saka, Abla Séfako Akakpo, Julienne Noude Téclessou, Abas Mouhari-Toure, Garba Mahamadou, Piham Gnossike, Waguéna Gnassingbé, Aurel Abilogoun-Chokki, Adjalamotom Tawelessi, Koussake Kombaté, Palokinam Pitché

**Affiliations:** 1 Dermatology Unit, Sylvanus Olympio Teaching Hospital, University of Lomé, B.P, 30785 Lomé, Togo; 2 Dermatology Unit, Campus Teaching Hospital, University of Lomé, Lomé, Togo; 3 Dermatology Unit, Kara Teaching Hospital, University of Kara, Kara, Togo

**Keywords:** Acne, Quality of life (QOL), Lomé (Togo)

## Abstract

**Background:**

Acne is a chronic inflammatory condition affecting the pilosebaceous follicle that mainly affects adolescents and young adults. The aim of this study was to assess the quality of life (QOL) of patients with acne, and to determine the correlation between the QOL and the severity of acne, in Lomé (Togo).

**Method:**

From July 2017 to February 2018, we conducted a study in three dermatology departments of Lomé. The clinical evaluation of acne and assessment of the QOL were done using the ECLA (*Echelle de Cotation des Lésions d’acné)* and CADI (Cardiff Acne Disability Index) scores respectively.

**Results:**

We enrolled 300 patients aged 12 to 52 years; 71.3% of whom were female. The face was affected by acne in 100% of cases and papulopustular acne was the most common clinical form (66.7%). Acne was mild to moderate in 162 patients (54%) and severe in 138 (46%). Impairment was observed in all patients’ QOL (scores ranged from 1 to 14 points). There was a positive correlation between severity of acne and QOL impairment in the patients (*r* = 0.21; *p* = 0.0002). We also found a positive correlation between overall CADI score and factors F1 and F3 of the ECLA scale: the severity of facial acne (*r* = 0.15; *p* = 0.0073) and the presence of scars (*r =* 0.21; *p* = 0.0002). In contrast, the global ECLA score was significantly correlated with items 2, 3, and 5 of the CADI questionnaire: the patient’s relationship (*r* = 0.13; *p* = 0.0241), avoidance behaviors (*r =* 0.21; *p* = 0.0002) and perception of acne (*r* = 0.16; *p* = 0.0067).

**Conclusion:**

Acne negatively impacts the QOL of patients. The severity of acne has an impact on the patient’s relationships, avoidance behaviors and perception of the acne.

**Electronic supplementary material:**

The online version of this article (10.1186/s12895-018-0075-z) contains supplementary material, which is available to authorized users.

## Background

Acne is a chronic inflammatory condition affecting the pilosebaceous follicle, involving four elements: the sebaceous gland, the follicular canal epithelium, a bacterium called *Propionibacterium acnes* and innate cutaneous immunity [1, 2]. Acne is a benign condition that mainly affects adolescents and young adults, whose prevalence varies according to the authors between 80 and 95% [3–7]. Despite this benign nature, many studies have shown that it has a negative impact on the quality of life (QOL) of patients [8–11], but so far, no such study has been conducted in Togo. The only study on acne published in Togo described the clinical features in patients using skin-bleaching products [12]. The aim of our study was to assess the QOL of patients with acne, and to determine the correlation between the QOL and the severity of acne in Togo.

## Method

We conducted a descriptive cross-sectional study between July 2017 and February 2018, in three dermatology units in Lomé (Togo). We included, after obtaining their consent, all patients diagnosed with acne by a dermatologist in these centers during the study period. Patients were recruited during the dermatology consultations. All the patients included in the study were visited for their acne for the first time and they were not already on treatment. The socio-demographic characteristics (age, sex, profession) were collected by a dermatologist during the interview and the physical examination. The dermatologist specified on the patient’s record the type of lesions, their location and the clinical form. The clinical evaluation of acne and QOL was performed with the ECLA (*Echelle de Cotation des lésions d’acné*) scale [13] (Additional file 1) and CADI (Cardiff Acne Disability Index) scale respectively [14] (Additional file 2). The two scales both French were validated in Togo par the Togolese Dermatology Society. The ECLA scale is composed of 3 factors (F1, F2 and F3) which respectively evaluate the type and intensity of facial acne, the extension and intensity of acne on regions other than the face and the presence of scars. The CADI questionnaire contained 5 items that respectively evaluate the emotions of the patient, his social relations, the avoidance behaviors, feelings of anxiety and the overall perception of acne. After each consultation, patients included in the study were asked to complete these two questionnaires.

### Calculation and interpretation of scores

The total ECLA score, obtained from the sum of the F1, F2, and F3 scores, ranged from 0 to 36. An ECLA score of 12 or less represented mild to moderate acne, a score greater than 12 represented severe acne. To establish the CADI score, the answers were graded as follows: (a) 3 points, (b) 2 points, (c) 1 point and (d) 0 point. The score calculated by adding the number of points for each question varied between 0 and 15. The interpretation was as follows: 0 = no change in QOL; 1 to 5 = slight impairment of QOL; 6 to 10 = moderate impairment of QOL; 11 to 15 = severe impairment of QOL.

### Data analysis

The data were entered using the software Epidata version 3.1. Before being analyzed, data had been verified and cleared. The statistical analyses were performed with the software R Studio version 3.3.4. The analysis initially enabled the description of the study population. The results were expressed in terms of relative and absolute frequency for the qualitative variables; mean and standard deviation for the quantitative variables. Then, for the quantitative variables, we used the Pearson correlation test or the Student’s t-test (two-way variables) to analyze the variability of the dependent variables according to the characteristics of the study population. For qualitative variables, we used the Analysis of variance method (ANOVA) for the variables with more than two modalities. The threshold of significance was set at 0.05.

## Results

**Fig. 1:**
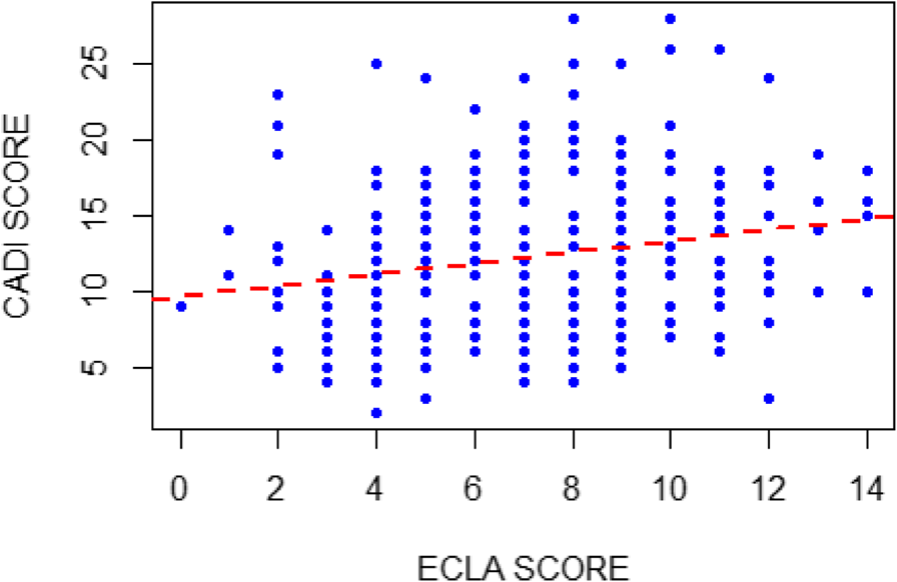
Correlation between ECLA and CADI scores (*r* = 0.21; *p* = 0.0002)

During the study period, 300 patients with acne were included. Their mean age was 23.7 ± 5.7 years (range: 12 to 52 years) and the sex-ratio (Male/Female) was 0.4. More than two-thirds (69.3%) of patients were less than 25 years old and the students were mostly represented (58.7%). The acne was located on the face in 100% of cases, alone or in combination with other locations. The most common acne lesions were papules (97.3%); papulopustular acne was the most common clinical form (66.7%) (Table 1). The mean ECLA severity score was 12.3 ± 5.0 (range: 2 to 28). Acne was mild to moderate in 162 patients (54.0%) and severe in 138 (46.0%). The mean CADI score was 7.3 ± 3.0 (range: 1 to 14). Impaired QOL was observed in all patients. It was considered mild in 85 patients, moderate in 166 and severe in 49. There was a positive correlation between the scores of the two scales ECLA and CADI (*r* = 0.21; *p* = 0.0002) (Fig. 1). A positive correlation was equally found between the overall CADI score and factors F1 and F3 of the ECLA scale (Table 2), as well as between the ECLA score and items 2, 3, and 5 of the CADI questionnaire (Table 3).

**Table 1:** Patient characteristics

Patient characteristics	Number	Percent
Type of lesions		
*Papules*	*292*	97.3
*Hyperseborrhoea*	*277*	92.3
*Blackheads*	*272*	90.7
*Hyperpigmented macules*	*249*	83.0
*Pustules*	*217*	72.3
*Excoriation*	*177*	59.0
*Crusty stitches*	*120*	40.0
*Punctuate depressions*	*104*	34.7
*Nodules*	*95*	31.7
*Cysts*	*83*	27.7
*Atrophic scars*	*33*	11.0
*Hypertrophic scars*	*23*	7.7
Location of lesions		
*Face*	*300*	100
*Back*	*190*	63,3
*Neck*	*140*	46,6
*Chest*	*136*	45,3
*Arms*	*60*	20,0
Clinical forms of acne		
*Papulo pustular acne*	*200*	66,7
*Retentional acne*	*58*	19,3
*Nodular acne*	*35*	11,7
*Pigmented acne*	*7*	2,3

**Table 2:** Correlation between CADI score and the three factors of the ECLA scale

	ECLA		
	Factor 1	Factor 2	Factor 3
CADI score (r)	0.15	0.11	0.21
*p-value*	*0.0073*	*0.0606*	*0.0002*

**Table 3:** Correlation between global ECLA score and the five items of the CADI score

	CADI				
	Item1	Item2	Item3	Item4	Item5
ECLA score (r)	0.05	0.13	0.21	0.08	0.16
*p-value*	*0.35*	*0.0241*	*0.0002*	*0.1640*	*0.0067*

## Discussion

The results of our study showed that i) 46% of patients had severe acne; ii) QOL of all patients was impaired; iii) There was a positive and significant correlation between severity of acne and impairment of patients’ QOL.

We found that the mean age of our patients was 23 ± 5.7 years, which was slightly less than those reported by other studies carried out in hospitals: 25 years in Cameroon [8], 25.6 years in Senegal [15] and 23.8 years in Morocco [16]. This relatively higher age reported in these studies could be due to the fact that in Togo, acne is neglected in adolescents since parents hardly seek medical care because it is considered as a normal phenomenon. In the study conducted in Burkina Faso, [17], the mean age of patients with acne was 19.5 years old, since the study was carried out in schools. We noted a female predominance like in the study of Kouotou et al. [8]. This is explained by the fact that women are more likely to seek medical care than men for any apparent and unsightly illness (100% of patients had facial lesions).

In our study, papulopustular acne was the most represented clinical form as in other series [8, 15]. We found 7 cases (2.3%) of acne pigmentosa, a form peculiar to black or pigmented skin. The frequency of excoriations (59%) could be related to self-injury likely triggered by obsessive disorders that acne causes.

We also found that the proportion of severe acne is high (46%) with a mean ECLA score of 12.3 ± 5. Slightly lower average ECLA scores were reported by Kouotou et al. in Cameroon (11 ± 4.5) [8] and Dreno et al. in France (8.26 ± 3.32) [9]. The proportion of severe forms found by Kouotou et al. [8] was also high (30.4%). On the other hand, the study conducted by Ouedraogo et al. [17] in Burkina Faso reported 4.75% of severe acne. This difference could be explained by the distinct settings of both studies, Ouedraogo et al. [17] having done theirs in schools.

In our study, QOL impairment was observed in all patients. Thus, even minimal acne can have a negative impact on QOL. The overall mean CADI score was 7.3 ± 3.0. This result is close to those of other studies that found mean values of 6.3 ± 3.4 [8], 4.86 ± 3.11 [18] and 4.8 ± 2.9 [19]. Many studies using other scales have also demonstrated QOL impairment in all patients [20, 21]. This proves that acne negatively affects the patient’s QOL, self-esteem and mood because of its apparent and unsightly nature.

In our study, the correlation between global ECLA and overall CADI was positive, suggesting that the more acne is severe, the more it will alter patients’ QOL. Apart from Dréno et al. [9], several studies [8, 17] found that there was a positive correlation between severity of acne and QOL impairment. These differences may be because these studies focused on different categories of populations. Other studies using different tools have also shown a relationship between the severity of acne and the QOL of the patients [10, 21]. Moreover, as in the study of Dréno et al. [9], we found a positive correlation between the CADI score and factors F1 and F3, meaning that the type and intensity of facial acne and the presence of scars influence QOL impairment of patients. There was no correlation between the CADI score and the F2 factor, (the location of acne lesions on regions other than the face) because this other location is hidden. However, Kouotou et al. [8] found a positive correlation between the CADI score and all three factors on the ECLA scale.

In contrast, there was a positive correlation between the severity of acne and patient relations (*p* = 0.0241), avoidance behaviors (*p* = 0.0002), and overall perception of acne (*p* = 0.0067). On the other hand, the severity of acne did not affect either the mood or the feelings of anxiety triggered by acne. This correlation between the ECLA score and the 5 items of CADI questionnaire was very different from one study to another [8, 9, 17]. While Kouotou et al. [8] found a positive correlation between ECLA and the 5 items, Ouedraogo et al. found a correlation between ECLA and items 2 and 4 [17]; while Dreno et al. [9] reported a correlation between ECLA and item 5. Of these four studies, correlations between ECLA and items 2 and 5 were mentioned three times, while correlations between ECLA and item 4 were mentioned twice. These four studies showed that the severity of acne is more correlated with the overall perception of acne, avoidance behaviors and feelings of anxiety triggered by acne.

### Limitations

The main limitation of this study was that it was conduct in hospital setting. Indeed, the detrimental effects of acne on a patient’s quality of life (relations, perception of acne) must have prompted a consultation. Secondly, no control group was used in this study. The use of a control group would have made it possible to see if the QOL impairment in the patient’s was really related to the presence of acne.

## Conclusion

Acne had a negative impact on the QOL of patients who suffer from it. The type and intensity of facial acne, and the presence of scars impacted the patient’s overall QOL. In addition, the overall severity of acne has an impact on the patient’s relationships, avoidance behaviors and overall perception of acne.

## Additional files

Additional file 1: Presentation of the ECLA scale. (DOCX 18 kb)

Additional file 2: Cardiff Acne Disability Index (CADI). (DOCX 15 kb)

## Abbreviations

ANOVA: Analysis of variance method
CADI: Cardiff acne disability index
ECLA: *Echelle de Cotation des Lésions d’Acné*
QOL: Quality of life
