# Supplementary material for: Acne in Lomé, Togo: clinical aspects and quality of life of patients
Source: BMC Dermatol. 2018 Aug 22;18:7. doi: 10.1186/s12895-018-0075-z (PMC6106930; doi:10.1186/s12895-018-0075-z)
Supplement: Supplementary file 1 — Presentation of the ECLA scale. (DOCX 18 kb) [file 12895_2018_75_MOESM1_ESM.docx]

**Additional file 1 Presentation of the ECLA scale**

| Factor 1 (F1) : Type and intensity ; Count on the whole face  Absent Rare Low Moderate Important Very important F1    = 0 = 1 = 2 = 3 = 4 = 5 |
| --- |
| None <5 5 to 9 10 to 19 20 to 40 >40  R Open and closed comedones  (Microcysts) R    Is Papules and None <5 5 to 9 10 to 19 20 to 40 > 40  pustules Is  Ip Nodules and None 1-2 3 4 5 > 5  Inflammatory cysts Ip |
| Score 1 = R+ Is + Ip |
| Factor 2 (F2) : Extension and intensity of acne; off the face |
| 0 1 3 4  Absent Low Moderate Important F2    Neck (C) High cervical area C  Low cervical area  Chest (P) P  Back (D) Sus scapula D  Under scapula  Arms (B) B |
| Score 2 = C+ P + D + B |
| Factor 3 (F3) : Absence of scars=0 ; Presence of scars=1 |
| Inflammatory Non-inflammatory Excoriations  CI CNI E |
| Score 3 = CI+ CNI + E |
| Final Score : Score 1 + Score 2 + Score 3 = |
| The ECLA score is therefore between 0 and 36 |
