# Supplementary material for: Acne in Lomé, Togo: clinical aspects and quality of life of patients
Source: BMC Dermatol. 2018 Aug 22;18:7. doi: 10.1186/s12895-018-0075-z (PMC6106930; doi:10.1186/s12895-018-0075-z)
Supplement: Supplementary file 2 — Cardiff Acne Disability Index (CADI). (DOCX 15 kb) [file 12895_2018_75_MOESM2_ESM.docx]

**Additional file 2 Cardiff Acne Disability Index (CADI)**

The scoring of each answer is as follows:

(a) 3

(b) 2

(c) 1

(d) 0

**Item 1**: In the past month, because of your acne pimples, have you been aggressive, frustrated or embarrassed? a. greatly; b. A lot; c. A little; d. Not at all

**Item 2**: In the last month, do you think your acne pimples affected your daily life, your parties or your relationship with your friend? a. significantly, in all my activities; b. Moderately, in most of my activities; c. Occasionally, or only in certain activities; d. Not at all

**Item 3**: In the past month, have you avoided public places or group outings because of your acne pimples? a. All the time; b. Most of the time; c. occasionally; d. Not at all

**Item 4**: In the last month, how does your skin feel? a. Feeling of anguish; b. Feelings of permanent concern; c. Feeling of occasional anxiety; d. No worries

**Item 5**: Can you tell how you perceive your acne today? a. A catastrophic problem; b. A major problem; c. A minor problem; d. Not a problem
